# Neurofibromatosis type 1, presenting as a rare widespread neurofibromas with cord compression

**Published:** 2016-01-05

**Authors:** Alireza Vakilian, Amir Moghadam-Ahmadi, Habib Farahmand

**Affiliations:** 1 Geriatric Care Research Center, Rafsanjan University of Medical Sciences, Rafsanjan, Iran; 2 Clinical Research Development Center, Rafsanjan University of Medical Sciences, Rafsanjan, Iran; 3 Department of Radiology, Ali-Ebn Abitaleb Hospital, Rafsanjan University of Medical Sciences, Rafsanjan, Iran

**Keywords:** Neurofibromatosis type 1, Neurofibroma, Spinsl Cord Compression

Neurofibromatosis type 1 (NF1) is a genetic disorder that disturbs cell growth in nervous system, causing tumors to form on nerve tissue. 

A 26-year-old man was brought to Ali-Ebn Abitaleb Hospital, Rafsanjan, Iran, with asymmetric quadriplegia more in left side. He was a known case of NF1. He first had a paraparesia two weeks before admission that progressed to quadriparesia. There was no family history of neurofibromatosis.

Hyperpigment lesions were revealed in variable sizes. Multiple nodular lesions and inguinal freckles were seen on his body surface. Babinski sign, hyperreflexia and spasticity were detected in lower limbs examination. Decreased sensation was revealed more in left side. Sphincter function was normal. In cervical spine magnetic resonance imaging (MRI), there were multiple mass lesions in paraspinal, intradural extramedullary and extradural region with cord compression from C1 to C6. Several neurofibromas were extraordinary in orbital cavity, neck, whole paraspinal tissues, mediastinum, para-aortic region, presacral, and pelvis with widening of neural foramina (dumbbell shaped lesions), also in perianal and sacral region (Figures 1-3).

Three months later, he was admitted to the surgery ward for an extensive laminectomy and reconstruction procedures.

As the most common symptom in other reports, our presenting case symptom was quadriparesis but asymmetric. In our case, there was multi-level involvement of cervical cord, not only C2 or C3 as in most reported cases.^1^

In a retrospective study on 97 patients with NF1, spinal curvature abnormality was present in 50/97 patients.^1^ In another case report, a 43-year-old man with history of NF1 presented with signs and symptoms of myelopathy and severe cervical kyphotic deformity.^2^ Our case was unique according to absence of deformity with widespread and plexiform neurofibromas.

In literature, there were some reported extensive neurofibromas that were less widespread than ours; and also there were 9 reported cases between the age of 3 to 15 years old with voluminous plexiform neurofibromas in neck region that were not as widespread as our case.^3^

**Figure 1:**
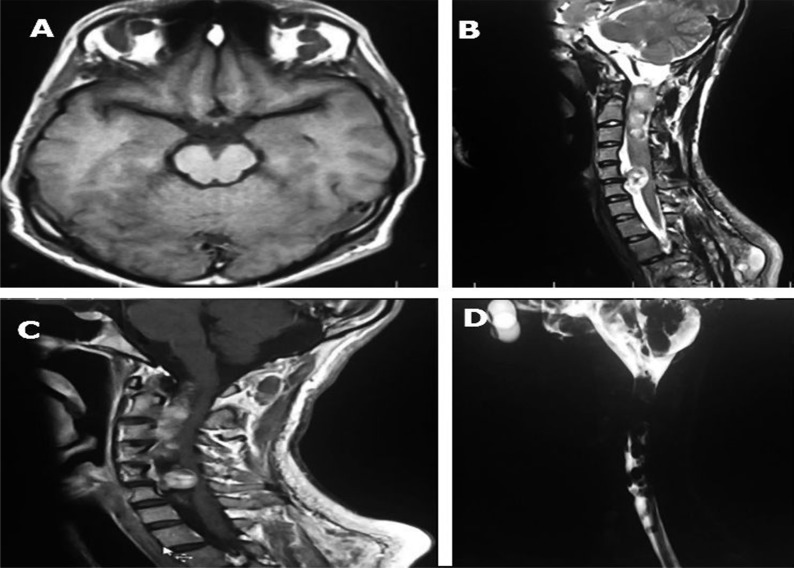
Widespread neurofibromas of orbital and cervical spine. Axial noncontrast T1-weighted image of brain reveals bilateral supra-orbital neurofibromas (A), Sagittal T2-weighted magnetic resonance imaging (MRI) of cervical spine reveals multiple intradural extramedullary neurofibromas, causing cord compression, widening of upper thoracic neural foramen and subcutaneous neurofibromas (B), Sagittal post-contrast T1-weighted image of cervical spine demonstrates multiple enhancing intradural extramedullary lesions causing cord compression (C), Sagittal cervical magnetic resonance myelogram ‎shows multiple intradural filling defects (D).

**Figure 2:**
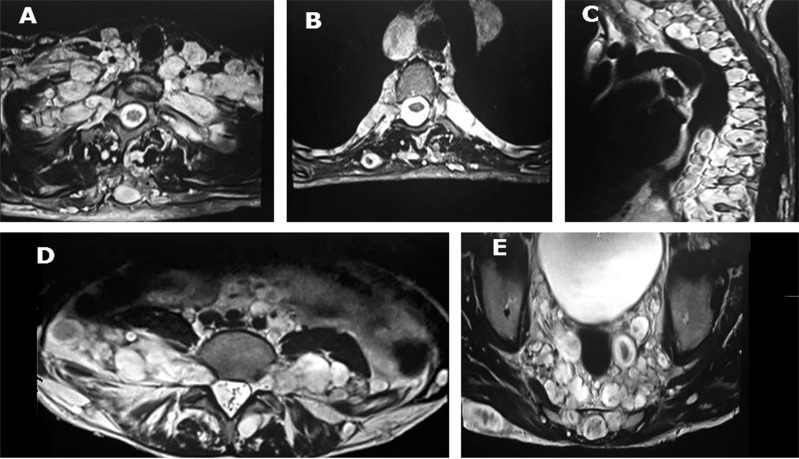
Widespread neurofibromas in neck, thorax, abdomen and pelvis; Axial T2-weighted image demonstrates widespread plexiform neurofibromas in the neck (A), Axial and parasagittal T2-weighted magnetic resonance imagings (MRI) demonstrate widespread cervical and thoracic paravertebral and mediastinal neurofibromas arising from cervicothoracic nerve roots (B and C), Axial T2-weighted MRI demonstrates multiple neurofibromas in the lumbar paravertebral, neural foraminal, para-aortic and retroperitoneal regions. Anterior displacement of bilateral psoas muscles is seen (D), Axial T2-weighted MRI demonstrate widespread pelvic neurofibromas in the perirectal and presacral spaces (E).

**Figure 3:**
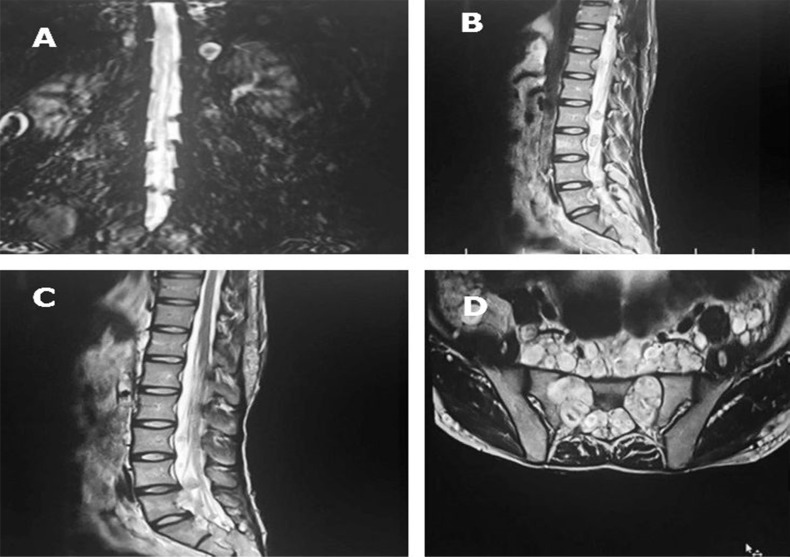
Widespread neurofibromas in lumbosacral nerve roots; In coronal magnetic resonance myelogram, multiple intradural neurofibromas arising from lumbosacral nerve roots are seen (A), Sagittal T2- weighted magnetic resonance imaging (MRI) of lumbosacral spine shows neurofibromas arising from nerve roots (B and C), Axial T2-weighted MRI demonstrates innumerable neurofibromas in the presacral space and in the sacral neural foramina causing enlargement of corresponding neural foramina (D).

Our case had diffuse involvement from orbital to sacral region and such extent of involvement has not been reported in related articles about patients with neurofibromatosis.

## Notes:

**How to cite this article:** Vakilian A, Moghadam-Ahmadi A, Farahmand H. Neurofibromatosis type 1, presenting as a rare widespread neurofibromas with cord compression. Iran J Neurol 2016; 15(1): 57-9.
